# Supplementary material for: Analysis of genetically driven alternative splicing identifies FBXO38 as a novel COPD susceptibility gene
Source: PLoS Genet. 2019 Jul 3;15(7):e1008229. doi: 10.1371/journal.pgen.1008229 (PMC6634423; doi:10.1371/journal.pgen.1008229)
Supplement: S1 Table — (DOCX) [file pgen.1008229.s001.docx]

**Supplementary Table 1: Clinical characteristics of COPDGene study individuals included in the analysis.**

|  | **Overall (n=376)** |
| --- | --- |
| Gender (% male) | 53.2 |
| Age, mean (SD) | 67.2 (8.4) |
| Current Smokers (% smokers) | 34.8 |
| Pack-Years Smoked, mean (SD) | 47.3 (23.5) |
| FEV1 percent predicted, mean (SD) | 73.6 (27.2) |
| Control - GOLD 0 (%)^1^ | 40.4 |
| COPD - GOLD 1 (%) | 9.8 |
| COPD - GOLD 2 (%) | 24.5 |
| COPD - GOLD 3 (%) | 16.2 |
| COPD - GOLD 4 (%) | 8.2 |
| Neutrophil percentage, mean (SD) | 61.8 (9.5) |
| Lymphocyte percentage, mean (SD) | 26.8 (8.6) |
| Eosinophil percentage, mean (SD) | 2.6 (2.2) |
| Monocyte percentage, mean (SD) | 8.2 (2.4) |

^1^Three individuals lack GOLD stage status due to lack of pulmonary function testing measurements
