# Supplementary material for: Analysis of genetically driven alternative splicing identifies FBXO38 as a novel COPD susceptibility gene
Source: PLoS Genet. 2019 Jul 3;15(7):e1008229. doi: 10.1371/journal.pgen.1008229 (PMC6634423; doi:10.1371/journal.pgen.1008229)
Supplement: S2 Table — (DOCX) [file pgen.1008229.s002.docx]

**Supplementary Table 2: cis eQTLs and sQTLs identified at the 10% FDR**

|  | **Cis-eQTL analysis** | **Cis-sQTL analysis** |
| --- | --- | --- |
| Tests conducted | 99,035,498 | 365,901,394 |
| Significant SNP-gene/intron pairs | 1,242,993 | 1,706,704 |
| Significant unique eQTL/sQTL SNPS | 708,928 | 561,060 |
| Significant genes/exons | 15,913 genes | 30,333 exons / 6742 genes |
