# Supplementary material for: Analysis of genetically driven alternative splicing identifies FBXO38 as a novel COPD susceptibility gene
Source: PLoS Genet. 2019 Jul 3;15(7):e1008229. doi: 10.1371/journal.pgen.1008229 (PMC6634423; doi:10.1371/journal.pgen.1008229)
Supplement: S3 Table — (DOCX) [file pgen.1008229.s003.docx]

**Supplementary Table 3: Number of introns with start and stop sites that are annotated vs. cryptic.**

| Verdict | Annotated to Genes^1^ |
| --- | --- |
| Fully Annotated | 18,951 |
| Cryptic 5’ splice site | 3,795 |
| Cryptic 3’ splice site | 3,986 |
| Cryptic Unanchored | 1,771 |
| Novel Annotated pair | 1,830 |

^1^Introns for which a most likely gene could be identified based on intron start and stop sites.
