# Supplementary material for: Analysis of genetically driven alternative splicing identifies FBXO38 as a novel COPD susceptibility gene
Source: PLoS Genet. 2019 Jul 3;15(7):e1008229. doi: 10.1371/journal.pgen.1008229 (PMC6634423; doi:10.1371/journal.pgen.1008229)
Supplement: S4 Table — (DOCX) [file pgen.1008229.s004.docx]

**Supplementary Table 4: KEGG pathways that are enriched in genes regulated by sQTLs but not eQTLs at the 10% FDR**

| Pathway | Number of genes supporting pathway | P-value | Bonferroni adjusted P-value |
| --- | --- | --- | --- |
| RNA transport | 44 | 5.70E-272 | 1.73E-269 |
| Endocytosis | 48 | 7.53E-125 | 2.28E-122 |
| Ubiquitin mediated proteolysis | 36 | 6.58E-122 | 1.99E-119 |
| Protein processing in endoplasmic reticulum | 36 | 2.52E-73 | 7.64E-71 |
| Lysosome | 27 | 5.36E-51 | 1.62E-48 |
| NOD-like receptor signaling pathway | 34 | 2.01E-33 | 6.08E-31 |
| RNA degradation | 18 | 1.93E-32 | 5.85E-30 |
| Proteasome | 14 | 3.49E-32 | 1.06E-29 |
| Autophagy - animal | 31 | 3.02E-31 | 9.14E-29 |
| Epstein-Barr virus infection | 41 | 9.17E-31 | 2.78E-28 |
| mTOR signaling pathway | 26 | 2.13E-28 | 6.46E-26 |
| Alzheimer's disease | 31 | 5.99E-24 | 1.82E-21 |
| Autophagy - other | 9 | 2.05E-19 | 6.21E-17 |
| Peroxisome | 15 | 6.92E-15 | 2.10E-12 |
| mRNA surveillance pathway | 18 | 9.86E-14 | 2.99E-11 |
| Cysteine and methionine metabolism | 9 | 7.39E-12 | 2.24E-09 |
| Herpes simplex infection | 33 | 1.21E-11 | 3.68E-09 |
| Aminoacyl-tRNA biosynthesis | 13 | 1.22E-11 | 3.70E-09 |
| Cellular senescence | 24 | 3.07E-11 | 9.29E-09 |
| Viral carcinogenesis | 31 | 1.45E-10 | 4.39E-08 |
| Platelet activation | 23 | 6.42E-10 | 1.95E-07 |
| Jak-STAT signaling pathway | 30 | 9.02E-10 | 2.73E-07 |
| Spliceosome | 20 | 3.77E-09 | 1.14E-06 |
| SNARE interactions in vesicular transport | 9 | 4.73E-09 | 1.43E-06 |
| N-Glycan biosynthesis | 10 | 2.02E-08 | 6.11E-06 |
| Hepatitis C | 27 | 6.13E-08 | 1.86E-05 |
| Neurotrophin signaling pathway | 20 | 1.72E-06 | 0.0005209 |
| Oxidative phosphorylation | 25 | 2.87E-06 | 0.0008705 |
| Tuberculosis | 32 | 1.02E-05 | 0.003091 |
| Lysine degradation | 11 | 1.39E-05 | 0.004197 |
| Mitophagy - animal | 13 | 2.08E-05 | 0.00629 |
| Adherens junction | 14 | 2.36E-05 | 0.007139 |
| Non-alcoholic fatty liver disease (NAFLD) | 29 | 2.60E-05 | 0.007875 |
