# Supplementary material for: Analysis of genetically driven alternative splicing identifies FBXO38 as a novel COPD susceptibility gene
Source: PLoS Genet. 2019 Jul 3;15(7):e1008229. doi: 10.1371/journal.pgen.1008229 (PMC6634423; doi:10.1371/journal.pgen.1008229)
Supplement: S5 Table — (DOCX) [file pgen.1008229.s005.docx]

**Supplementary Table 5: Reactome pathways that are enriched in genes regulated by sQTLs but not eQTLs at the 10% FDR**

| Pathway | Number of genes supporting pathway | P-value | Bonferroni adjusted P-value |
| --- | --- | --- | --- |
| DNA Repair | 61 | 5.99E-60 | 6.22E-57 |
| Antigen processing: Ubiquitination & Proteasome degradation | 49 | 6.40E-56 | 6.64E-53 |
| COPII-mediated vesicle transport | 22 | 3.46E-47 | 3.59E-44 |
| Processing of Capped Intron-Containing Pre-mRNA | 51 | 1.24E-40 | 1.28E-37 |
| Translation | 60 | 8.08E-35 | 8.38E-32 |
| Signaling by Receptor Tyrosine Kinases | 86 | 1.36E-33 | 1.41E-30 |
| Respiratory electron transport | 22 | 1.60E-32 | 1.66E-29 |
| Processing of SMDT1 | 8 | 1.63E-28 | 1.69E-25 |
| Asparagine N-linked glycosylation | 64 | 2.39E-27 | 2.48E-24 |
| Neddylation | 36 | 7.69E-27 | 7.98E-24 |
| RNA polymerase II transcribes snRNA genes | 19 | 1.14E-26 | 1.19E-23 |
| ER to Golgi Anterograde Transport | 36 | 6.25E-23 | 6.49E-20 |
| COPI-mediated anterograde transport | 21 | 5.33E-22 | 5.53E-19 |
| PPARA activates gene expression | 23 | 2.34E-21 | 2.43E-18 |
| Mitochondrial translation initiation | 23 | 2.97E-21 | 3.08E-18 |
| RAB GEFs exchange GTP for GDP on RABs | 17 | 1.49E-19 | 1.55E-16 |
| Cargo recognition for clathrin-mediated endocytosis | 23 | 2.04E-19 | 2.12E-16 |
| E3 ubiquitin ligases ubiquitinate target proteins | 13 | 4.20E-18 | 4.36E-15 |
| Signaling by BRAF and RAF fusions | 13 | 5.58E-18 | 5.79E-15 |
| SUMO E3 ligases SUMOylate target proteins | 39 | 4.21E-17 | 4.37E-14 |
| FGFR2 alternative splicing | 9 | 6.34E-17 | 6.58E-14 |
| mRNA Splicing - Major Pathway | 36 | 9.33E-17 | 9.69E-14 |
| Gene and protein expression by JAK-STAT signaling after Interleukin-12 stimulation | 13 | 1.88E-16 | 1.95E-13 |
| Deubiquitination | 43 | 7.87E-15 | 8.17E-12 |
| SUMOylation of DNA damage response and repair proteins | 21 | 3.28E-14 | 3.41E-11 |
| DNA Damage Recognition in GG-NER | 12 | 8.68E-14 | 9.01E-11 |
| Clathrin-mediated endocytosis | 31 | 1.31E-13 | 1.36E-10 |
| Downregulation of ERBB2 signaling | 7 | 1.82E-13 | 1.89E-10 |
| Retrograde transport at the Trans-Golgi-Network | 13 | 1.06E-12 | 1.10E-09 |
| Organelle biogenesis and maintenance | 53 | 1.28E-12 | 1.33E-09 |
| SUMOylation of RNA binding proteins | 17 | 1.42E-12 | 1.47E-09 |
| NIK-->noncanonical NF-kB signaling | 5 | 2.57E-12 | 2.67E-09 |
| Cytosolic sensors of pathogen-associated DNA | 16 | 2.69E-12 | 2.79E-09 |
| Nuclear Pore Complex (NPC) Disassembly | 12 | 6.03E-12 | 6.26E-09 |
| Peroxisomal protein import | 12 | 1.22E-11 | 1.26E-08 |
| UCH proteinases | 13 | 2.01E-11 | 2.08E-08 |
| rRNA modification in the nucleus and cytosol | 14 | 1.11E-10 | 1.15E-07 |
| RUNX1 interacts with co-factors whose precise effect on RUNX1 targets is not known | 12 | 1.46E-10 | 1.51E-07 |
| Deadenylation of mRNA | 8 | 5.11E-10 | 5.30E-07 |
| table(t$downstream)VEGFA-VEGFR2 Pathway | 20 | 5.29E-10 | 5.49E-07 |
| DNA Double-Strand Break Repair | 25 | 6.54E-10 | 6.79E-07 |
| Host Interactions of HIV factors | 25 | 6.65E-10 | 6.91E-07 |
| RORA activates gene expression | 8 | 7.54E-10 | 7.83E-07 |
| Intra-Golgi and retrograde Golgi-to-ER traffic | 36 | 9.80E-10 | 1.02E-06 |
| HIV Infection | 34 | 1.41E-09 | 1.46E-06 |
| SUMOylation | 40 | 2.46E-09 | 2.55E-06 |
| Macroautophagy | 16 | 3.11E-09 | 3.23E-06 |
| TP53 Regulates Metabolic Genes | 21 | 6.13E-09 | 6.36E-06 |
| M Phase | 53 | 8.09E-09 | 8.40E-06 |
| Signaling by SCF-KIT | 11 | 9.38E-09 | 9.74E-06 |
| Diseases of signal transduction | 54 | 1.70E-08 | 1.77E-05 |
| tRNA processing in the nucleus | 17 | 3.14E-08 | 3.26E-05 |
| Late Phase of HIV Life Cycle | 28 | 4.80E-08 | 4.99E-05 |
| Transcriptional regulation by small RNAs | 14 | 7.79E-08 | 8.08E-05 |
| ER Quality Control Compartment (ERQC) | 6 | 1.13E-07 | 0.0001177 |
| Formation of ATP by chemiosmotic coupling | 5 | 1.16E-07 | 0.0001204 |
| Anchoring of the basal body to the plasma membrane | 15 | 1.21E-07 | 0.0001256 |
| Major pathway of rRNA processing in the nucleolus and cytosol | 31 | 1.68E-07 | 0.0001745 |
| Cargo concentration in the ER | 11 | 8.21E-07 | 0.0008518 |
| Mitochondrial translation elongation | 22 | 9.89E-07 | 0.001026 |
| Mitochondrial translation termination | 22 | 9.89E-07 | 0.001026 |
| InlB-mediated entry of Listeria monocytogenes into host cell | 4 | 1.35E-06 | 0.001404 |
| Intra-Golgi traffic | 11 | 1.87E-06 | 0.001941 |
| Purine ribonucleoside monophosphate biosynthesis | 4 | 1.90E-06 | 0.001976 |
| Mitochondrial biogenesis | 21 | 2.05E-06 | 0.00213 |
| VxPx cargo-targeting to cilium | 6 | 2.15E-06 | 0.002233 |
| The role of Nef in HIV-1 replication and disease pathogenesis | 11 | 2.35E-06 | 0.002439 |
| Gene Silencing by RNA | 21 | 6.17E-06 | 0.006407 |
| DARPP-32 events | 8 | 6.83E-06 | 0.007089 |
| Toll Like Receptor 9 (TLR9) Cascade | 16 | 8.29E-06 | 0.008601 |
