# Supplementary material for: Analysis of genetically driven alternative splicing identifies FBXO38 as a novel COPD susceptibility gene
Source: PLoS Genet. 2019 Jul 3;15(7):e1008229. doi: 10.1371/journal.pgen.1008229 (PMC6634423; doi:10.1371/journal.pgen.1008229)
Supplement: S2 Fig — (DOCX) [file pgen.1008229.s010.docx]

**Supplementary Figure 2: Schematic diagram illustrating the generation of splice clusters and calculation of splice ratios for 5q32**


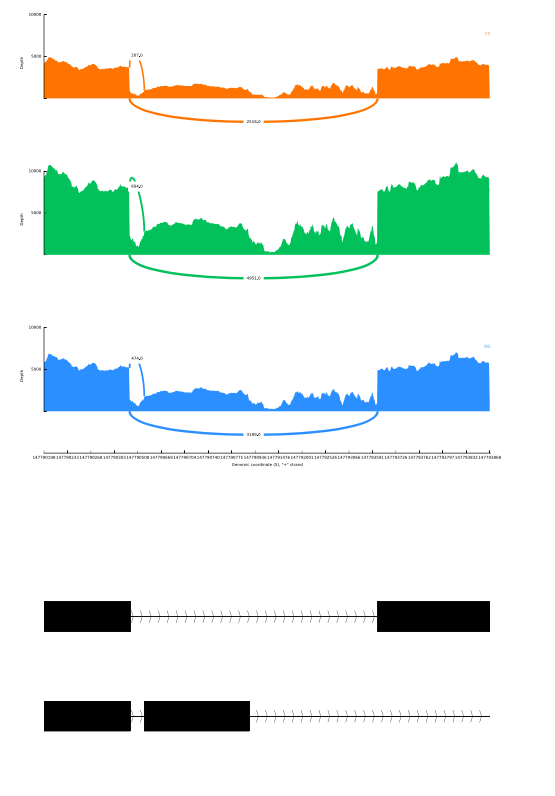

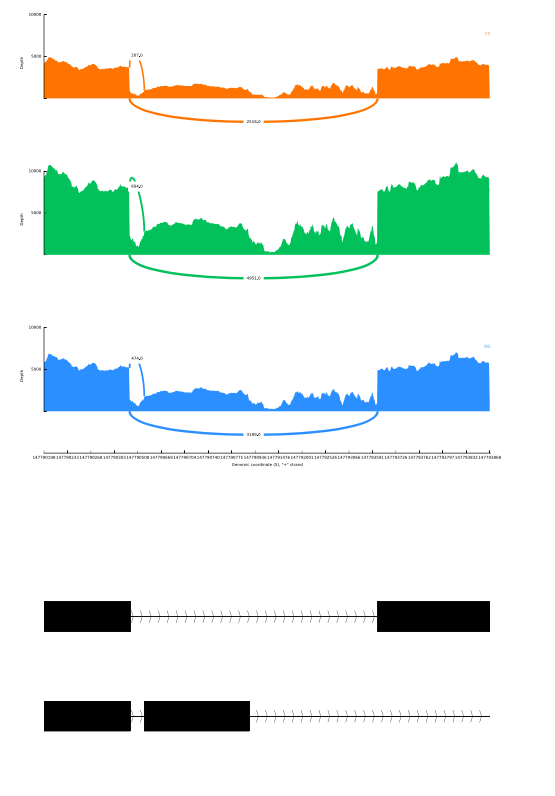


Splice site 1

Splice site 3

Splice site 2

Exon 9

Exon 10

Exon 9

Exon 10

Novel Exon

FBXO38 Isoforms

RNASeq reads mapped to genome

Cluster reads according to shared intron start and stop positions

Cluster clu_10408

Splice site 1

Calculate splice ratios

Splice site 2

Splice site 3

Splice site 1

Splice site 1 + 2 + 3

Splice site 2

Splice site 1 + 2 + 3

Splice site 3

Splice site 1 + 2 + 3

RNA-seq reads are mapped to the genome, and reads align to both previously annotated as well as undocumented exons. Using leafcutter, reads are clustered together based on shared start and stop intron positions. Splice ratios are then calculated by dividing the number of reads that support the presence of a given junction by the total number of reads in the cluster. This ratio is used as the input for
