# Supplementary material for: Analysis of genetically driven alternative splicing identifies FBXO38 as a novel COPD susceptibility gene
Source: PLoS Genet. 2019 Jul 3;15(7):e1008229. doi: 10.1371/journal.pgen.1008229 (PMC6634423; doi:10.1371/journal.pgen.1008229)
Supplement: S3 Fig — (DOCX) [file pgen.1008229.s011.docx]

**Supplementary Figure 3: Enrichment of low P-value associations with COPD case control status among sQTL and eQTL SNPs at the 10% FDR**


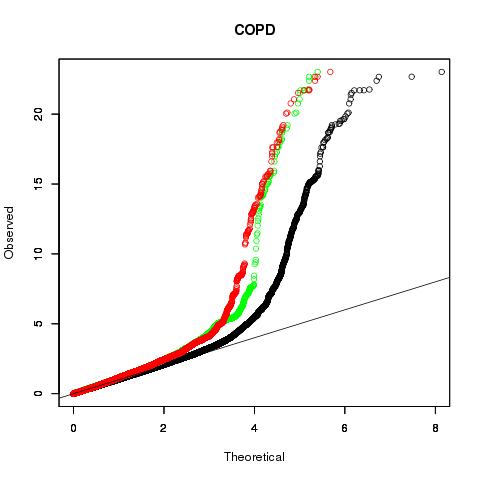


sQTLs (558,660)

eQTLs (706,030)

Genome-wide

COPD Case/Control GWAS data

-log10 p-value

-log10 p-value

**Q-Q plot showing observed vs. expected**

1. **–GWAS –log10 p-values for genome wide GWAS data (black),**
2. **GWAS –log10 p-values for SNPs that are also sQTLs (red),**
3. **GWAS –log10 p-values for SNPs that are also eQTLs (green).**

**sQTLs appear more highly enriched in GWAS associations with low p-value.**
