# Supplementary material for: Analysis of genetically driven alternative splicing identifies FBXO38 as a novel COPD susceptibility gene
Source: PLoS Genet. 2019 Jul 3;15(7):e1008229. doi: 10.1371/journal.pgen.1008229 (PMC6634423; doi:10.1371/journal.pgen.1008229)

**Supplementary Figure 5: FBXO38 interacts with Cullin 1, but not other Cullin family members in 293T cells.**


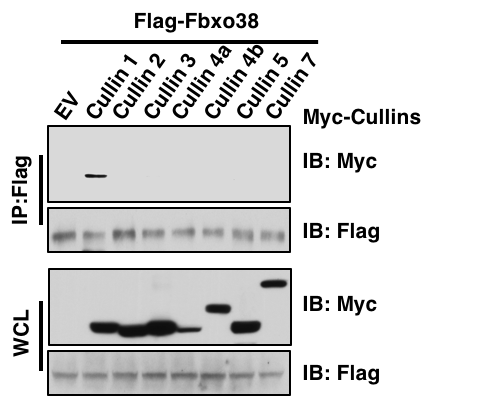

Supplement: S5 Fig — (DOCX) [file pgen.1008229.s013.docx]
