# Supplementary material for: Analysis of genetically driven alternative splicing identifies FBXO38 as a novel COPD susceptibility gene
Source: PLoS Genet. 2019 Jul 3;15(7):e1008229. doi: 10.1371/journal.pgen.1008229 (PMC6634423; doi:10.1371/journal.pgen.1008229)
Supplement: S6 Fig — (DOCX) [file pgen.1008229.s014.docx]

**Supplementary Figure 6: rs4788084 is associated with a splice site in SULT1A2 as well as SULT1A2 whole gene expression**
