# Supplementary material for: Analysis of genetically driven alternative splicing identifies FBXO38 as a novel COPD susceptibility gene
Source: PLoS Genet. 2019 Jul 3;15(7):e1008229. doi: 10.1371/journal.pgen.1008229 (PMC6634423; doi:10.1371/journal.pgen.1008229)
Supplement: S1 Text — (DOCX) [file pgen.1008229.s016.docx]

**Acknowledgements:**

**COPDGene Investigators – Core Units**

*Administrative Center*: James D. Crapo, MD (PI); Edwin K. Silverman, MD, PhD (PI); Barry J. Make, MD; Elizabeth A. Regan, MD, PhD

*Genetic Analysis Center*: Terri Beaty, PhD; Ferdouse Begum, PhD; Robert Busch, MD; Peter J. Castaldi, MD, MSc; Michael Cho, MD; Dawn L. DeMeo, MD, MPH; Adel R. Boueiz, MD; Marilyn G. Foreman, MD, MS; Eitan Halper-Stromberg; Nadia N. Hansel, MD, MPH; Megan E. Hardin, MD; Lystra P. Hayden, MD, MMSc; Craig P. Hersh, MD, MPH; Jacqueline Hetmanski, MS, MPH; Brian D. Hobbs, MD; John E. Hokanson, MPH, PhD; Nan Laird, PhD; Christoph Lange, PhD; Sharon M. Lutz, PhD; Merry-Lynn McDonald, PhD; Margaret M. Parker, PhD; Dandi Qiao, PhD; Elizabeth A. Regan, MD, PhD; Stephanie Santorico, PhD; Edwin K. Silverman, MD, PhD; Emily S. Wan, MD; Sungho Won

*Imaging Center*: Mustafa Al Qaisi, MD; Harvey O. Coxson, PhD; Teresa Gray; MeiLan K. Han, MD, MS; Eric A. Hoffman, PhD; Stephen Humphries, PhD; Francine L. Jacobson, MD, MPH; Philip F. Judy, PhD; Ella A. Kazerooni, MD; Alex Kluiber; David A. Lynch, MB; John D. Newell, Jr., MD; Elizabeth A. Regan, MD, PhD; James C. Ross, PhD; Raul San Jose Estepar, PhD; Joyce Schroeder, MD; Jered Sieren; Douglas Stinson; Berend C. Stoel, PhD; Juerg Tschirren, PhD; Edwin Van Beek, MD, PhD; Bram van Ginneken, PhD; Eva van Rikxoort, PhD; George Washko, MD; Carla G. Wilson, MS;

*PFT QA Center, Salt Lake City, UT*: Robert Jensen, PhD

*Data Coordinating Center and Biostatistics*, *National Jewish Health, Denver, CO*: Douglas Everett, PhD; Jim Crooks, PhD; Camille Moore, PhD; Matt Strand, PhD; Carla G. Wilson, MS

*Epidemiology Core*, *University of Colorado Anschutz Medical Campus, Aurora, CO*: John E. Hokanson, MPH, PhD; John Hughes, PhD; Gregory Kinney, MPH, PhD; Sharon M. Lutz, PhD; Katherine Pratte, MSPH; Kendra A. Young, PhD

**COPDGene Investigators – Clinical Centers**

*Ann Arbor VA:* Jeffrey L. Curtis, MD; Carlos H. Martinez, MD, MPH; Perry G. Pernicano, MD

*Baylor College of Medicine, Houston, TX*: Nicola Hanania, MD, MS; Philip Alapat, MD; Mustafa Atik, MD; Venkata Bandi, MD; Aladin Boriek, PhD; Kalpatha Guntupalli, MD; Elizabeth Guy, MD; Arun Nachiappan, MD; Amit Parulekar, MD;

*Brigham and Women’s Hospital, Boston, MA*: Dawn L. DeMeo, MD, MPH; Craig Hersh, MD, MPH; Francine L. Jacobson, MD, MPH; George Washko, MD

*Columbia University, New York, NY*: R. Graham Barr, MD, DrPH; John Austin, MD; Belinda D’Souza, MD; Gregory D.N. Pearson, MD; Anna Rozenshtein, MD, MPH, FACR; Byron Thomashow, MD

*Duke University Medical Center, Durham, NC*: Neil MacIntyre, Jr., MD; H. Page McAdams, MD; Lacey Washington, MD

*HealthPartners Research Institute, Minneapolis, MN*: Charlene McEvoy, MD, MPH; Joseph Tashjian, MD

*Johns Hopkins University, Baltimore, MD*: Robert Wise, MD; Robert Brown, MD; Nadia N. Hansel, MD, MPH; Karen Horton, MD; Allison Lambert, MD, MHS; Nirupama Putcha, MD, MHS

*Los Angeles Biomedical Research Institute at Harbor UCLA Medical Center, Torrance, CA*: Richard Casaburi, PhD, MD; Alessandra Adami, PhD; Matthew Budoff, MD; Hans Fischer, MD; Janos Porszasz, MD, PhD; Harry Rossiter, PhD; William Stringer, MD

*Michael E. DeBakey VAMC, Houston*, *TX*: Amir Sharafkhaneh, MD, PhD; Charlie Lan, DO

*Minneapolis VA:* Christine Wendt, MD; Brian Bell, MD

*Morehouse School of Medicine, Atlanta, GA*: Marilyn G. Foreman, MD, MS; Eugene Berkowitz, MD, PhD; Gloria Westney, MD, MS

*National Jewish Health, Denver, CO*: Russell Bowler, MD, PhD; David A. Lynch, MB

*Reliant Medical Group, Worcester, MA*: Richard Rosiello, MD; David Pace, MD

*Temple University, Philadelphia, PA:* Gerard Criner, MD; David Ciccolella, MD; Francis Cordova, MD; Chandra Dass, MD; Gilbert D’Alonzo, DO; Parag Desai, MD; Michael Jacobs, PharmD; Steven Kelsen, MD, PhD; Victor Kim, MD; A. James Mamary, MD; Nathaniel Marchetti, DO; Aditi Satti, MD; Kartik Shenoy, MD; Robert M. Steiner, MD; Alex Swift, MD; Irene Swift, MD; Maria Elena Vega-Sanchez, MD

*University of Alabama, Birmingham, AL:* Mark Dransfield, MD; William Bailey, MD; Surya Bhatt, MD; Anand Iyer, MD; Hrudaya Nath, MD; J. Michael Wells, MD

*University of California, San Diego, CA*: Joe Ramsdell, MD; Paul Friedman, MD; Xavier Soler, MD, PhD; Andrew Yen, MD

*University of Iowa, Iowa City, IA*: Alejandro P. Comellas, MD; John Newell, Jr., MD; Brad Thompson, MD

*University of Michigan, Ann Arbor, MI*: MeiLan K. Han, MD, MS; Ella Kazerooni, MD; Carlos H. Martinez, MD, MPH

*University of Minnesota, Minneapolis, MN*: Joanne Billings, MD; Abbie Begnaud, MD; Tadashi Allen, MD

*University of Pittsburgh, Pittsburgh, PA*: Frank Sciurba, MD; Jessica Bon, MD; Divay Chandra, MD, MSc; Carl Fuhrman, MD; Joel Weissfeld, MD, MPH

*University of Texas Health Science Center at San Antonio, San Antonio, TX*: Antonio Anzueto, MD; Sandra Adams, MD; Diego Maselli-Caceres, MD; Mario E. Ruiz, MD

**ICGC Executive Committee**: James Crapo, William MacNee, David Lynch, Dirkje Postma, Edwin Silverman, Jorgen Vestbo. *Members*: Alvar Agusti, Wayne Anderson, Nawar Bakerly, Per Bakke, Robert Bals, Kathleen Barnes, R. Graham Barr, Terri Beaty, Eugene Bleecker, Marike Boezen, Yohan Bosse, Russell Bowler, Christopher Brightling, Marleen de Bruijne, Guy Brusselle, Peter Castaldi, Bartolome Celli, Michael Cho, Harvey Coxson, Ron Crystal, Dawn DeMeo, Asger Dirksen, Jennifer Dy, Marilyn Foreman, Judith Garcia-Aymerich, Pierre Gevenois, Hester Gietema, Ian Hall, Nadia Hansel, Craig Hersh, Eric Hoffman, John Hokanson, Pim de Jong, Phil Judy, Noor Kalsheker, Hans-Ulrich Kauczor, Woo Jin Kim, Lies Lahousse, Tarja Laitinen, Diether Lambrechts, Sang Do Lee, Augusto Litonjua, David Lomas, Stephanie London, Daan Loth, Sharon Lutz, Masaharu Nishimura, John Newell, Merry-Lynn McDonald, Borge Nordestgaard, George O'Connor, Yeon-Mok Oh, Peter Pare, Massimo Pistolesi, Milo Puhan, Elizabeth Regan, Stephen Rennard, Stephen Rich, Andrew Sandford, Joon-Beom Seo, Andrea Short, Martijn Spruit, Berend Stoel, David Strachan, Gerben ter Riet, Nicola Sverzellati, Yohannes Tesfaigzi, Martin Tobin, Edwin Van Beek, Bram van Ginneken, Claus Vogelmeier, Adam Wanner, George Washko, Els Wauters, Emiel Wouters, Robert Young, and Loems Zeigler-Heitbrock

**References**

1. Li YI, Knowles DA, Humphrey J, Barbeira AN, Dickinson SP, Im HK, Pritchard JK. Annotation-free quantification of RNA splicing using LeafCutter. *Nat Genet* 2017.

2. Hormozdiari F, van de Bunt M, Segre AV, Li X, Joo JWJ, Bilow M, Sul JH, Sankararaman S, Pasaniuc B, Eskin E. Colocalization of GWAS and eQTL Signals Detects Target Genes. *Am J Hum Genet* 2016; 99: 1245-1260.

3. Morrow JD, Zhou X, Lao T, Jiang Z, DeMeo DL, Cho MH, Qiu W, Cloonan S, Pinto-Plata V, Celli B, Marchetti N, Criner GJ, Bueno R, Washko GR, Glass K, Quackenbush J, Choi AM, Silverman EK, Hersh CP. Functional interactors of three genome-wide association study genes are differentially expressed in severe chronic obstructive pulmonary disease lung tissue. *Sci Rep* 2017; 7: 44232.

4. Foroushani AB, Brinkman FS, Lynn DJ. Pathway-GPS and SIGORA: identifying relevant pathways based on the over-representation of their gene-pair signatures. *PeerJ* 2013; 1: e229.
